# Effectiveness and equity of the Tanzania National Voucher Scheme for mosquito nets over 10 years of implementation

**DOI:** 10.1186/s12936-017-1902-0

**Published:** 2017-06-15

**Authors:** Karen Kramer, Renata Mandike, Rose Nathan, Ally Mohamed, Matthew Lynch, Nick Brown, Ally Mnzava, Wilhelmina Rimisho, Christian Lengeler

**Affiliations:** 1 Swiss Tropical & Public Health Institute, P.O. Box 4002, Basel, Switzerland; 2 University of Basel, Petersplatz 1, 4003 Basel, Switzerland; 3 National Malaria Control Programme, Ministry of Health, Community Development, Gender, Elderly and Children, Dar es Salaam, Tanzania; 4 Ifakara Health Institute, Dar es Salaam, Tanzania; 5 Johns Hopkins Bloomberg School of Public Health, Baltimore, USA; 6 A to Z Textile Mills Ltd, Arusha, Tanzania

**Keywords:** Public private partnership, Malaria, Vector control, Insecticide-treated nets, Long-lasting insecticidal nets, Keep up strategy, Continuous distribution, Tanzania

## Abstract

**Background:**

The Tanzania National Voucher Scheme (TNVS) was a public private partnership managed by the Ministry of Health that provided pregnant women and infants with highly subsidized (long-lasting) insecticide-treated nets between 2004 and 2014. It was implemented in the context of the National Insecticide Treated Nets (NATNETS) Programme and was the main keep up strategy for vulnerable populations.

**Case description:**

The programme design was adjusted considerably over time to incorporate new evidence, shifting public health policies, and changing donor priorities. Three TNVS models can be distinguished: (1) the fixed discount; (2) the fixed top-up; (3) the hybrid voucher model. The changes improved equity and effectiveness, but also had a profound effect on how the programme was managed and implemented.

**Results:**

The TNVS reached the majority of beneficiaries with vouchers, and significantly increased household ownership and use of LLINs. While two mass distribution campaigns implemented between 2009 and 2011 achieved universal coverage and equity, the TNVS ensured continuous protection of the vulnerable populations before, during and after the campaigns. The TNVS stimulated and maintained a large national retail network which managed the LLIN supply chain.

**Discussion and lessons learned:**

The effectiveness of the TNVS was a function of several interdependent factors, including the supply chain of vouchers through the public health system; the supply chain of nets in the commercial sector; the demand for nets from voucher recipients; management and risk mitigation measures; and the influence of global and donor objectives.

**Conclusion:**

The TNVS was a highly innovative and globally influential programme, which stimulated the thinking around effectively and equitably distributing ITNs, and contributed directly to the evolution of global policy. It was a fundamental component of the NATNETS programme which protected a malaria-vulnerable population for over a decade.

## Background

Since 2000, the Tanzanian Government has implemented the National Insecticide Treated Nets (NATNETS) Programme to scale up the distribution and use of insecticide-treated nets (ITNs) and, since 2009, long-lasting insecticidal nets (LLINs). LLINs are recognized as an effective primary prevention for malaria and the large-scale introduction of ITNs and LLINs has proceeded in most malaria endemic countries since 2004 [1]. Long-term protection with LLINs is dependent on achieving and then maintaining a high coverage level in the overall population. In practice, this requires two sets of integrated strategies: (1) a ‘Catch-Up’ strategy that allows a fast increase in the coverage of LLINs in the country, and which is usually achieved by free mass distribution campaigns; and (2) ‘Keep-Up’ strategies aimed at maintaining a high net coverage through the continuous provision of nets through appropriate channels (health facilities, schools, and the private sector) [2]. Both catch-up and keep-up strategies should be supported by Behaviour Change Communication Campaigns [3].

From 2004 until mid-2014, the Tanzanian National Voucher Scheme (TNVS) was the key distribution mechanism under NATNETS to increase access to and use of (long-lasting) insecticide-treated nets amongst pregnant women and young children. The TNVS provided these two target groups with a discount voucher during attendance at a reproductive and child health (RCH) facility. The vouchers could then be exchanged for an ITN or LLIN at a participating retail outlet at greatly reduced price. The voucher concept was initially developed in the frame of the Swiss KINET project (1996–2000), before being expanded nationally [4–6].

The TNVS was a public private partnership (PPP) under leadership of the Ministry of Health, and included multilateral and bilateral development partners, non-governmental organizations, academic institutions, mosquito net manufacturing companies, wholesalers and retailers. Funding for the programme was provided by: The Global Fund to Fight AIDS, Tuberculosis and Malaria (GFATM) from 2003 until 2011; the United States Agency for International Development (USAID) through the President’s Malaria Initiative (PMI) from 2006 until 2013; and the UK Department for International Development (DFID) from 2011 until 2014. The TNVS reached national coverage in 2006 and represents one of the largest and most enduring keep-up programmes targeting pregnant women and young children in any endemic country.

This report aims to comprehensively describe the functioning of the TNVS. It then examines the effectiveness and equity of the TNVS, considering the contextual factors leading to changes in the design of the programme.

## Case description

The TNVS provided two types of vouchers: a Pregnant Women Voucher (PWV) given to pregnant women at their first visit to an antenatal clinic; and an infant voucher (IV) introduced in November 2006 given to parents/caretakers of infants with their first measles vaccination. Both vouchers types were provided in paper form until 2011, and then in electronic form.

**Fig. 1:**
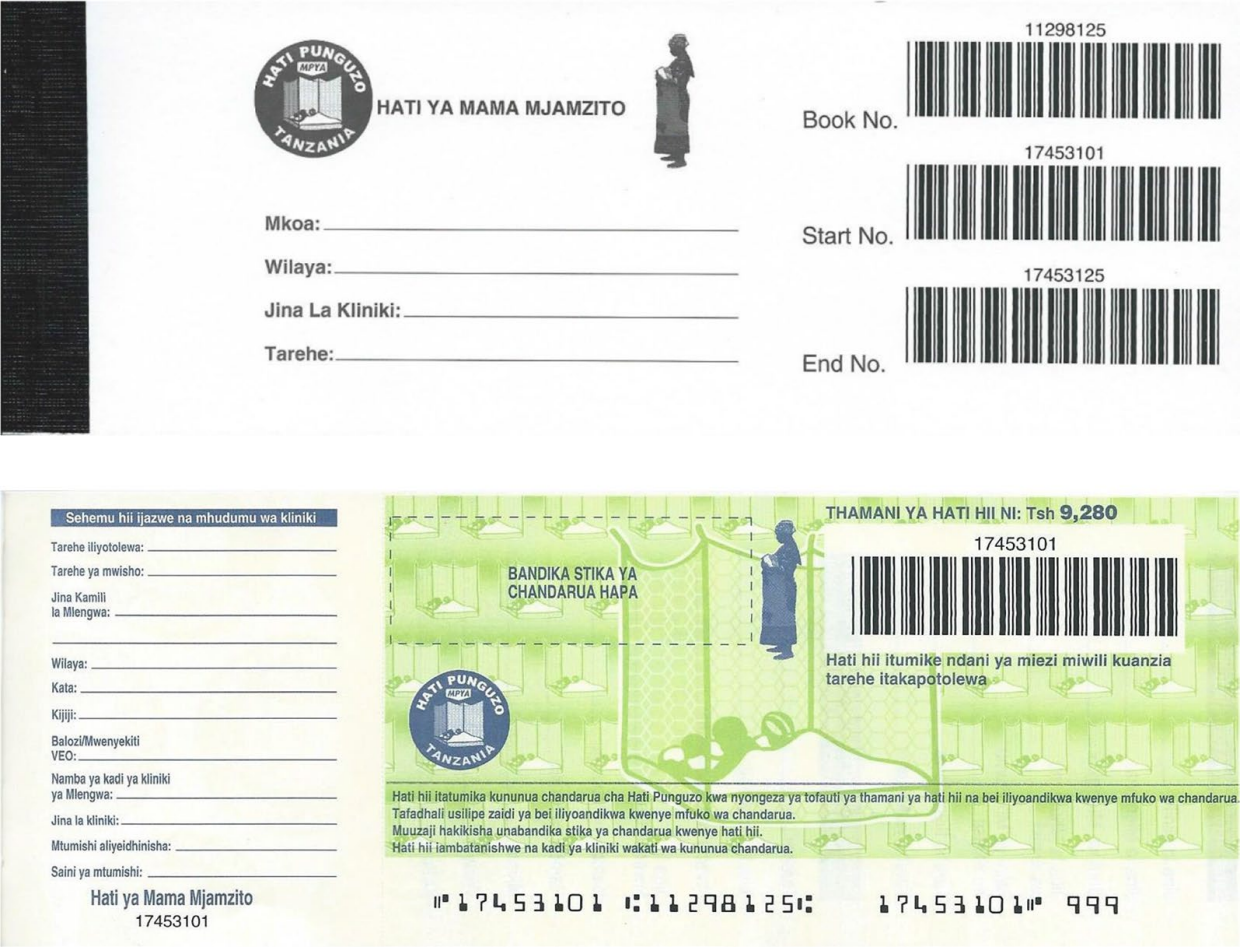
Example of a Pregnant Woman Voucher Booklet and Voucher

The paper vouchers followed a lengthy cycle from the Logistics Contractor of the TNVS to the District Medical Officers (DMOs), who distributed the voucher booklets to health facilities, who in turn issued vouchers to beneficiaries. Each voucher booklet contained fifty vouchers and was barcoded (Fig. 1). The date of dispatch as well as the name of the region, district, and health facility was recorded on the front of the booklet. The voucher stub and voucher had matching barcodes to enable tracking of where the voucher was issued and redeemed. The vouchers included security features to prevent forgery. The name of the health facility, village and ward, as well as the name of the beneficiary and the RCH card number were recorded on both the voucher and the stub. Later versions of the voucher also included a space for the barcoded sticker enclosed with the LLIN.

**Fig. 2:**
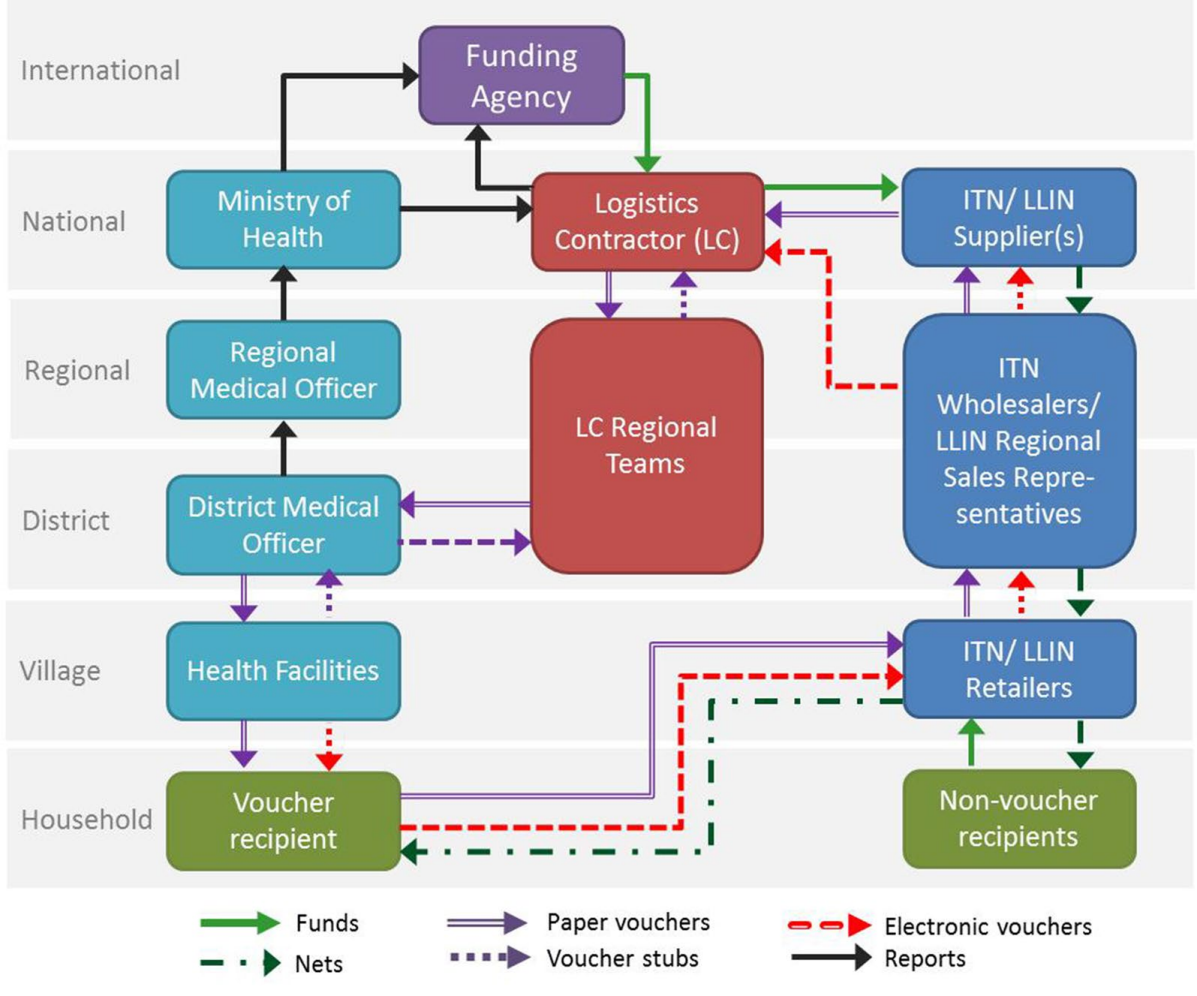
TNVS Flow Chart. See text for explanation of the processes

District Medical Officers collected the empty booklets with voucher stubs from health facilities and returned these to the Logistics Contractor. Retailers submitted their redeemed vouchers to the mosquito net suppliers, who submitted these to the Logistics Contractor. The barcodes on the voucher stubs and redeemed vouchers were then scanned and matched. The paper voucher cycle could take as long as 9 months. The electronic voucher was piloted in 2011 and rolled out in 2012 to improve efficiency of the voucher cycle. A flow chart of the TNVS is shown in Fig. 2. Funding flows varied per donor. The GFATM funding was channeled via the Ministry of Finance and the Ministry of Health to the Logistics Contractor, whereas PMI and DFID funded the Logistics Contractor directly.

The design of the TNVS evolved considerably over time based on new evidence, shifting public health policies, and changing donor priorities. Three distinct models can be distinguished during the lifetime of the programme, largely related to the way the value of the voucher was set: (1) fixed discount for ITNs (2004–2009); (2) fixed top-up for LLINs (2010–2012); and a hybrid voucher system (2013–2014).

### 2004–2009: fixed discount voucher for ITNs

#### Design and objectives

The TNVS was launched in 2004 and rolled out to all regions of Tanzania mainland by 2006. The aim was to increase coverage of ITNs to 60% amongst pregnant women and infants, in line with the targets set then in the Abuja Declaration in 2000 [7]. The initial model was embedded in the premise of achieving ITN upscaling through a strong commercial supply chain for nets supplemented by subsidized sales to target populations [6]. During this period, the voucher provided a *fixed discount* on a choice of four locally-produced brands of polyester nets bundled with an insecticide retreatment kit.

The implementation of the programme was done by contractors selected by the Ministry of Health through competitive bidding. Contractors included a Logistics Contractor (Mennonite Economic Development Associates), Training and Communication Contractors (World Vision Tanzania and Care International), and a Monitoring and Evaluation Contractor (Ifakara Health Research and Development Centre—now known as the Ifakara Health Institute, together with the London School of Hygiene and Tropical Medicine). The TNVS partnered with four existing Tanzanian net manufacturers (A–Z Textile Mills, Sunflag, Motex and TMTL) who worked through a network of wholesalers and retailers.

During this time, the TNVS closely collaborated with the SMARTNET project, a large social marketing project implemented by Population Services International between 2002 and 2007. The aim of SMARTNET was to increase commercial availability of nets at affordable prices, to establish a nationwide culture of net use, and to increase the percentage of treated nets [8]. SMARTNET provided free insecticide retreatment kits to the four Tanzanian net manufacturers to bundle with their brand of polyester nets, thereby turning them into treated nets. SMARTNET also implemented a nationwide generic campaign to promote ITN use, and provided the net manufacturers with specific marketing support. The project supported the development of a strong ITN retail network, particularly in urban and peri-urban areas.

#### Process

**Fig. 3:**
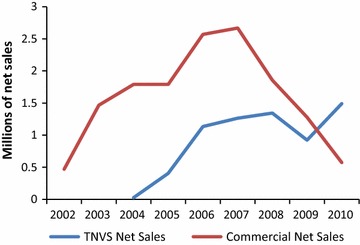
Commercial net sales compared to TNVS net sales (2002–2010)

The four net manufacturers supplied an initial stock of nets of varying sizes and shapes to wholesalers on credit. The flow of nets from manufacturer to wholesaler to retailers was based on a *pull*-*system* of vouchers being exchanged for new nets. A voucher recipient could exchange her voucher at any participating retail outlet. The manufacturer was reimbursed the amount of the voucher by the Logistics Contractor after the redeemed voucher had been matched to the corresponding stub. The top-up amount paid by the beneficiary constituted the profit margin for the retailer. The vouchers created a demand for nets countrywide, leading to a rapid expansion of the retail network into the rural areas. Supported by SMARTNET, volume of sales of both voucher nets and full-priced commercial nets increased rapidly throughout the country [9]. Figure 3 shows annual sales of unsubsidized ITNs from 2002 to 2010, compared to ITNs and LLINs bought with a voucher over the same period.

#### Challenges and modifications

The retail network made it possible for the nets to reach the grassroots level in both rural and urban areas. However, in remote under-populated areas retail prices were generally higher due to higher transport costs and lower demand. Stock-out of nets was also more likely [10]. Originally, the discount voucher had a value of 2750 Tanzanian Shillings (equivalent to approximately USD 2.40 at the time). The top-up amount paid by the voucher beneficiary varied depending on retail prices, ranging between TSH 700 (USD 0.60) to over TSH 2300 (USD 2.00). This led to decreased redemption rates, particularly amongst the lower socioeconomic groups [11]. To reduce the top up amount and compensate for increasing retail costs and dropping redemption rates the voucher value was raised to TSH 3250 (equivalent to US, D 2.90) in 2006 [12].

### 2009–2012: fixed top-up voucher for LLIN

#### Design and objectives

The TNVS, as the first national voucher programme, had both stimulated and informed global thinking about the role of the private sector in net distribution and in creating a sustainable commercial market. However, with changing global trends in malaria control the design and the objectives of the TNVS changed.

In 2008, the international target of 60% ITN coverage of vulnerable populations was expanded to 80% coverage of all populations at risk from malaria [13]. In order to rapidly ‘catch up’ to the new universal coverage target, the global malaria community through the Global Fund to Fight AIDS TB and Malaria (GFATM) made available the hundreds of millions of dollars required for mass distribution of free LLINs. Tanzania’s first mass campaign targeting children under 5 years was implemented in 2009–2010 and distributed 8.7 million nets. It was immediately followed by a second campaign from 2010 to 2011, targeting the remaining uncovered sleeping spaces which distributed 17.6 million nets [14].

The mass campaigns also marked the switch from ITNs to the more effective but costlier LLINs. The TNVS switched to LLINs several months after the start of the first mass campaign. As a result, the SMARTNET Project no longer provided free insecticide retreatment kits to the net manufacturers. This meant that all commercially sold polyester nets previously bundled with insecticide from then on became untreated nets. As LLINs were approximately three times the price of an ITN at the time, a *fixed top-up* voucher was introduced which reduced the top-up amount to be paid by pregnant women and mothers at TSH 500 (USD 0.30). The main reason for the change was to ensure equity in access amongst the voucher beneficiaries and to prevent price variation throughout the country. The role of the private sector as a distribution channel for TNVS nets remained, but the objective of stimulating and sustaining a viable commercial market for LLINs was no longer a priority.

#### Process

The fixed top-up voucher was redeemable for a single type and size of LLIN, which was not for sale commercially to non-voucher recipients. At the time, only one LLIN manufacturer was operating in Tanzania (A–Z Textiles Mills), which transformed the programme from a multi-supplier to a single-supplier model. The new model was based on a *push system* of the LLIN manufacturer supplying retail outlets directly, as opposed to the earlier model which was intended to be regulated through normal supply and demand market forces. The Logistics Contractor reimbursed the LLIN supplier for every redeemed voucher. The LLIN supplier restocked LLIN to the retailers for each voucher redeemed. The TSH 500 top-up paid by the beneficiary constituted revenue for the retailer, and hence their incentive to participate.

The switch to free distribution of LLINs was preceded by a heated international debate about whether free campaigns would cause the existing targeted subsidy models such as the TNVS to collapse [15]. This was later not found to be the case for the TNVS. The reduced top-up amount paid by beneficiaries after the introduction of the fixed top-up voucher for LLINs dramatically increased redemption rates amongst all socioeconomic groups, despite the fact that the introduction coincided with the roll out of the first mass campaign [16].

#### Challenges and modifications

The TNVS faced a number of challenges during this period. A key constraint in the paper voucher system was the frequent stock outs of voucher booklets at health facilities. This was largely due to facilities not returning the booklets with voucher stubs to the District Medical Officers, leading to a delay in restocking by the Logistics Contractor. This in turn caused great delays in payments to the LLIN supplier since redeemed vouchers had to be matched with the corresponding stub. DMOs also faced logistical constraints in distributing new voucher booklets to health facilities.

A second concern from the donor perspective was that the paper vouchers, which had been dispatched to DMOs but which had not yet been returned to the Logistics Contractor, represented a fiscal liability [17]. To address this issue, the donors set voucher liability targets, limiting the number of vouchers in circulation at any given time [18]. When targets were reached, voucher issuance would be stopped until existing vouchers were redeemed.

To alleviate the problem, two measures were taken to shorten the time that a voucher was in circulation. A 60-day voucher validity period was introduced in 2010 [19]. This was followed by the piloting and roll out of the electronic voucher in 2011. The e-voucher allowed for tracking at each step of the redemption process using mobile phones, SMS messaging and a web-based database. The electronic system provided real-time data on number of vouchers issued by RCH facilities and number of vouchers redeemed by retail outlets. This also enabled faster payment of vouchers to the supplier.

Another constraint during this time was that because of the higher purchase price of LLINs, many retailers lacked capital to purchase sufficient inventory of LLINs. As a result, stock-outs of LLINs occurred frequently. The programme, therefore, introduced seed capital agreements, which provided existing TNVS retailers an initial stock of ten free LLINs when the retailer purchased five additional LLINs with their own capital [20].

### 2013–2014: hybrid voucher model

#### Design and objectives

Although greatly improving voucher uptake and equity, the fixed top-up voucher model was not conducive to the re-development of a sustainable commercial market for LLINs. Recognizing this as a serious constraint, a hybrid voucher model was introduced in early 2013 with the aim to create the necessary conditions for commercial market development. This model was based on the following principles: (1) voucher recipients should be given more choice in terms of type, size, and shape of LLIN that they could buy with the voucher; (2) equity should be maintained and beneficiaries should be able to access a ‘base-net’ (similar size as the previous TNVS net) at the same fixed top-up price; (3) all LLINs included in the TNVS should also be commercially available at retail price to non-voucher customers in order to open up the market and make LLINs more widely available in all areas of Tanzania; and (4) fair competition among manufacturers should be encouraged in order to improve quality and reduce costs.

#### Process

The value of the new voucher was determined through a competitive process issued to eligible LLIN suppliers. Two suppliers (A–Z Textile Mills with Olyset^®^ and BestNet with NetProtect^®^) submitted bids. The new voucher value was set at TSH 9250 (USD 5.80) and the set retail price of the standard sized net at TSH 9750 (USD 6.10). The top-up price for the voucher recipient remained TSH 500 (USD 0.30) for a standard sized net but was slight more for a larger size net.

Performance-based contracting introduced by DFID in 2012 provided bonuses and penalties for the Logistics Contractor linked to voucher redemption targets and retail coverage [21]. The goal was to have one retailer within a 5-km radius of each Reproductive and Child Health facility. For better management, the country was divided into two zones: easy-to-reach (approximately 85% of the country) and hard-to-reach (15%). The two LLIN suppliers were required to sign a standard, non-exclusive contract with retailers in the easy-to-reach areas. The hard-to-reach areas were divided among the two suppliers. The suppliers were responsible for: ensuring retail availability of LLINs throughout the country; making provision for the temporary storage of nets prior to distribution to villages; and putting in place adequate logistical systems for the efficient and timely delivery [22].

The suppliers provided new stock of LLINs to their retailers in exchange for redeemed vouchers. The supplier was paid USD 5.80 by the Logistics Contractor for every properly redeemed voucher, with a distribution premium of USD 0.30 for vouchers from hard-to-reach areas. However, according to the suppliers, this premium only partially addressed the considerable additional delivery costs involved (estimated at USD 1.25 per net).

#### Challenges

For a number of reasons the hybrid voucher model never reached its full potential in terms of creating a commercial market for LLINs. A–Z Textile Mills Ltd. had a pre-existing retail network but the new LLIN supplier BestNet was delayed in building its own network, particularly in hard-to reach areas. Poor mobile phone connectivity in these areas also hampered enrollment of new retailers in the e-voucher mechanism. LLIN stock outs of both brands were frequent, particularly in the hard-to-reach areas [23]. Commercial sales of LLINs were almost non-existent as the demand for full-priced LLINs was low due to high retail prices. Retailers were also reluctant in stocking a variety of sizes, shapes and colours as it tied up working capital. Finally, retail margins were generally low.

In October 2013, the WHO Pesticide Evaluation Scheme (WHOPES) temporarily suspended its recommendation for the LLIN brand NetProtect^®^ supplied by BestNet. In 2014 WHOPES completely revoked the recommendation as the required field studies were found not to comply with the WHO requirement for testing and evaluation of LLINs [24]. Thus, BestNet dropped out of the programme and TNVS reverted back to a single-supplier model with almost all of the sales coming from vouchers.

### Closure of the TNVS

**Fig. 4:**
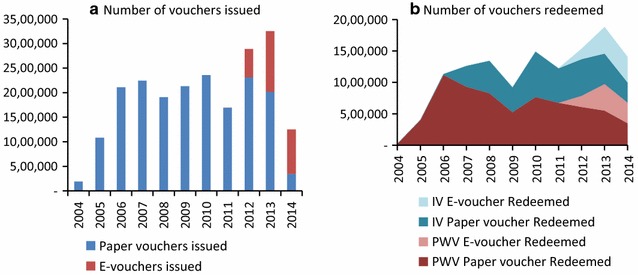
**a** Total vouchers issued per year and voucher type. **b** Total vouchers redeemed per year, per group and voucher type. *V* infant voucher, *PWV* Pregnant Women Voucher

In the last quarter of 2013 there was a big peak in absolute number of vouchers redeemed, with the highest quarterly redemption in the history of the programme (Fig. 4).

The increase was initially attributed to efforts by the Logistics Contractor to increase voucher redemption under the performance-based contract with DFID. However, further analysis of the TNVS regional data showed that during this period the quarterly redemption rates in a few regions greatly exceeded the expected numbers of beneficiaries [25]. Subsequently, the donor (DFID—at the time the sole funder of the TNVS) commissioned a special audit to investigate this unexpected development. The audit uncovered cases of fraudulent activities in the e-voucher mechanism by some clinic and retail staff. However, the audit report was not shared with the Ministry of Health and the full extent of the fraud was never made public.

Key to the fraud was that it entailed collusion between health facility staff, retail outlets, and sales representatives. In the electronic voucher system, the name of the beneficiary was not recorded. As there were no unique identifiers for RCH clinic cards, it was possible for health facility staff to create vouchers for fake beneficiaries, and collude with retailers to redeem these vouchers for nets. The problem was exacerbated by the fact that by 2013 7% of retail outlets were owned by health workers [26]. For every voucher redeemed by a fake beneficiary, new LLINs would be provided by the sales representatives and sold illicitly on the commercial market.

In addition to fraud, clinics were also found to be over-issuing infant vouchers, contrary to policy. In these cases, clinic staff issued multiple vouchers to the same infant during different vaccination visits and issued vouchers to non-targeted children (aged more than 1 year).

As a result of the audit findings, DFID cancelled its funding for the electronic voucher in mid-June 2014 and for the paper voucher mid-July 2014. The infant voucher had already been discontinued in May 2014, following a new agreement between the Ministry of Health and DFID. This decision was not related to the fraud findings but was reached to free funding to continue the pregnant woman voucher until the end of 2015, when PMI would have taken over funding again. However, following the audit, PMI no longer considered funding the TNVS.

## Results

This section presents results on three key outcomes of the TNVS, namely the extent to which the TNVS was effective in (1) improving access to ITNs/LLINs by the target populations; (2) increasing ownership and use of ITNs/LLINs by the target populations and; (3) stimulating the development of a commercial market for ITNs/LLINs.

Equity is measured through access and use of nets amongst all socio-economic groups and geographical settings. Socioeconomic status was constructed using principal components analysis. The indicators included in the index were a mixture of household ownership of assets, housing conditions and education of household head. The continuous variable produced by the principal components analysis was divided into five equal sized groups (quintiles) [27].

### Access to ITNs/LLINs

Access by the target population is a function of RCH attendance rates, number of vouchers issued to pregnant women and infants attending an RCH facility, and number of voucher recipients redeeming the voucher for a (long-lasting) insecticide-treated net. In short, it expresses the percentage of the eligible groups (pregnant women and infants) who redeemed the voucher in a shop against a net. All data on vouchers issued and redeemed come from the TNVS database maintained by the Logistics Contractor (Mennonite Economic Development Associates). In absence of reliable health facility data, pregnant women and infant populations have been calculated using nationally available crude birth rate, perinatal mortality rate and infant mortality rates per 1000 live births [28–31].

Figure 4 above shows the total number of vouchers issued and redeemed per year from 2004 to the closure of the TNVS in 2014. The number of paper vouchers issued to DMOs varied considerably per year and per donor. The total number of vouchers redeemed increased rapidly until 2007 as the TNVS was rolling out and grew steadily until 2008. Redemption numbers decreased significantly in 2009, due to increasing top-up amounts described above and increased again in 2010 after introduction of the fixed top up voucher. In 2011 numbers dropped again due to less vouchers being funded by PMI because of concerns regarding financial liability (described above). With the introduction of the e-voucher in 2012 redemption numbers of both the PWV and IV subsequently increased.

**Fig. 5:**
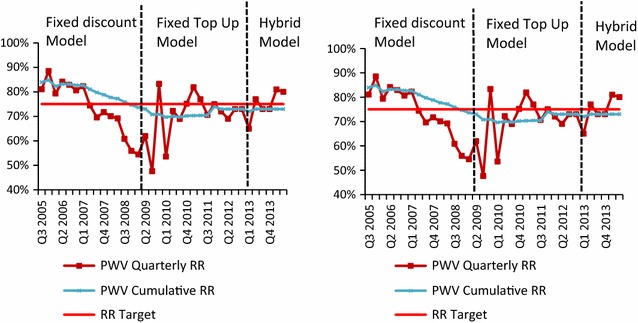
Quarterly and cumulative redemption rates for pregnant women and infant vouchers. **a** Pregnant Woman Voucher (PWV). **b** infant voucher (IV)

Redemption rates measure the proportion of vouchers used by the beneficiaries over a particular period. For paper vouchers, the redemption rate is defined as the total number of vouchers returned (with corresponding stub) within a quarter, divided by the total number of stubs returned within the same quarter. The combined quarterly redemption rates for both voucher types (shown in Fig. 5) declined from the early high levels when the top-up amount was generally considered affordable and fluctuated considerably until the introduction of the fixed top-up voucher in 2009. From January 2010 to June 2011 (during two mass campaigns), pregnant women voucher redemption rates returned to the initial level by rising from 54 to 77%, while infant voucher redemption rates increased from 51 to 81%, hence reaching values above the initial levels. Cumulative redemption rates for both the pregnant woman and infant voucher were 73 and 74%, respectively.

**Fig. 6:**
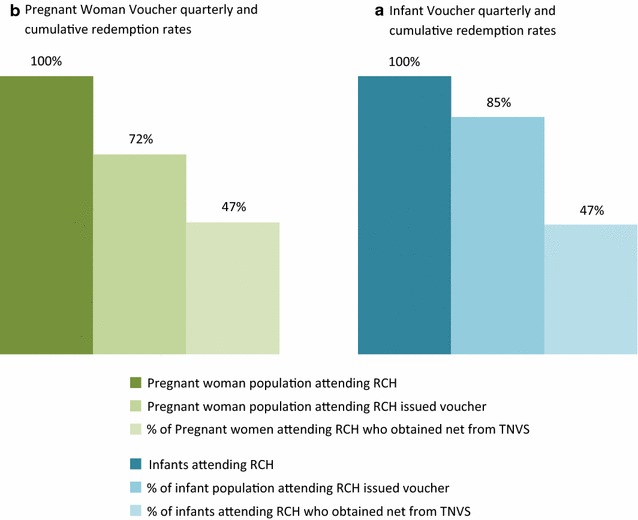
Proportion of pregnant woman and infant population attending an RCH clinic who were issued a voucher and used it to purchase an ITN/LLIN

Figure 6 shows the proportion of pregnant women and infants attending an RCH clinic that received a voucher and used it to purchase an ITN or LLIN. The rate is based on averages for the period 2006–2013 for pregnant women (after national roll out of the pregnant woman voucher and before closure) and for the period 2007–2013 for infants. RCH facility attendance was higher for pregnant women than for infants (95 and 83% respectively) [30, 31]. On average, 72% of the total pregnant woman population and 85% of the total infant population attending an RCH facility received a voucher. 47% of the pregnant women and 47% of infants who attended an RCH clinic accessed a net through the TNVS (constituting 45% of the total projected pregnant women population and 33% of the projected infant population).

Hence, clearly the proportion of the eligible population (defined as pregnant women and infants attending an RCH facility) who accessed an ITN/LLIN through the TNVS on an annual basis was considerably less than 100%, with the exception of the first quarter of 2014 when fraudulent activities were ongoing amongst a number of clinics and retailers in a number of regions.

### Household ownership and net use in Tanzania

The percentage of households with at least one ITN increased from 22.5% in 2004 to 38.3% in 2007 before implementation of the mass campaigns. This increased to 63.4% in 2010 after the first under-five catch-up campaign and to 91.5% in 2011 after the second mass campaign. The mean number of nets per household increased from less than one per household in 2004 to 2.5 in 2012. The equity ratio (ratio of ownership in the poorest households to the least poor) showed a dramatic improvement from 0.1 in 2004 to 0.3 in 2008 to unity by 2011 [30–33].

The proportion of pregnant women and children under 5 years sleeping under an ITN net increased from 15.4 and 15.9% in 2004 to 26 and 24.8% in 2008. Use increased significantly to 76.2 and 72.9% in 2012 after the two mass campaigns [30–33]. Before 2010, households in the wealthiest quintile were more likely to have slept under an ITN. This ratio equalized after the campaigns. Pregnant women and children under 5 years in urban areas were more likely to sleep under an ITN before the campaigns indicating a clear urban bias, which was corrected through the free mass distribution.

### A commercial market for ITNs and LLINs

The initial target for retailer coverage was one retailer in 90% of the villages accepting vouchers and selling ITNs [18]. During the hybrid model phase (2013–2014), the retailer target was amended to one retailer within a 5-km radius of each RCH facility, with special focus on hard-to-reach areas [34]. The number of participating retailers for the period 2007–2010 was approximately 6500, dropping to 5400 by 2011 during the second mass distribution campaign. During the period 2007–2010 the average retailer to clinic ratio was 1.5. It dropped to 1.1 in 2011 and gradually increased again to 1.4 [35]. However, the distribution of retailers was not evenly distributed amongst regions or amongst villages. Nationally representative TNVS retail audits showed a decrease in the proportion of retail outlets stocking ITNS/LLIN from 29% in 2008 to 6% in 2013 (after the introduction of the fixed discount voucher and after the two mass campaigns). By 2013 only 35% of villages had one or more net retailer compared to 44% in 2011 [36].

Sales data from the Tanzanian net manufacturers showed a significant increase of unsubsidized ITN sales between 2002 and 2007 [37], which were almost double that of TNVS sales during the same period. However commercial sales of ITNs decreased after the end of the SMARTNET Strategic Social Marketing Project in 2008. Untreated polyester nets continued to be sold but these sales decreased during the mass campaigns. 52% retailers indicated a decrease in commercial sales following the first mass campaign and 75% after the second mass campaign, with many established retailers leaving the business [36]. A commercial market for LLINs was never established for reasons described above.

## Discussion and lessons learned

The effectiveness of the TNVS was a function of several interdependent factors, including the supply chain of vouchers through the public health system; the supply chain of nets in the commercial sector; the demand for nets from voucher recipients; management and risk mitigation measures; and the influence of global and donor objectives.

### Voucher supply chain

The voucher supply chain was influenced by the functioning of the public health system, as well as the availability and continuity of funding. RCH attendance in Tanzania on average is high for both pregnant women and infants, so the potential reach of the TNVS was large. The main constraint was voucher stock outs at health facilities. Nevertheless, most the TNVS beneficiaries received a voucher during an RCH visit.

There is a clear correlation between number of vouchers issued and number of vouchers redeemed. Target setting for voucher distribution varied per donor, and since the TNVS was supported by several donors in turn, the targets consequently also varied. Limiting of paper voucher distribution by PMI in 2010 and 2011 caused significant voucher stock outs at heath facilities, resulting in retailers stocking less ITNs/LLINs. This led to a subsequent lag in voucher redemption [38]. On the other hand, performance-based contracting introduced by DFID in 2012 lead to a significant increase in total number of vouchers issued, particularly the electronic voucher.

### ITNs/LLINs supply chain

During the period of the fixed discount voucher model (2004–2009), the TNVS functioned on the basis of a partnership with four ITN suppliers who worked through a network of wholesalers and retailers. The initial assumptions were that a private sector distribution system was more efficient and cheaper than the delivery of physical nets through health facilities, and that a public private partnership helped create and sustain a ‘net culture’ that would outlast donor supported programmes. However, market forces were found not to be ideal to target hard-to-reach or poorer areas and did poorly on equity. Lower demand for nets (for various reasons including price and voucher availability) inevitably led to some retailers stocking fewer nets.

Under the fixed top-up voucher model (2009–2012), the one existing LLIN supplier had a price agreement with the TNVS and was responsible for managing the supply chain and retail network. As a result, the TNVS no longer played a stimulating function for the commercial market. It clearly also hindered other potential LLIN suppliers to enter the market. In effect, the LLIN supplier became a contractor to a donor-funded programme, providing only nets to voucher recipients. Maintaining sufficient LLIN stock in all the participating retail outlets proved problematic, with frequent LLIN stock outs, particularly in hard-to-reach areas. But despite the lack of demand for commercial nets, the TNVS did manage to maintain a national network of between 6000–7000 retailers throughout the duration of the programme.

### Demand factors

The major demand determinants of redemption rates were the top up amount paid by the beneficiaries, retailer to clinic ratio, and socioeconomic status [16]. In the first model (2004–2009), the TNVS played a key role in expanding the existing urban market for ITNs to peri-urban and rural areas, as it created demand for nets throughout the country [8]. In addition, the nets purchased with a voucher provided households an opportunity to test the nets without paying the full price, thereby acting as a market stimulant for future commercial sales [39]. The reduced top-up amount paid by beneficiaries after the introduction of the fixed discount voucher for LLINs (2009–2012) dramatically increased redemption rates amongst all socio-economic groups, even though the introduction coincided with the roll out of the first mass LLIN distribution campaign [16].

Cumulative redemption rates for both the pregnant woman and infant voucher was 73%, indicating a high demand for subsidized nets. According to a voucher tracking survey conducted in 2011, key barriers to exchanging a voucher for a net was lack of net stock at the outlet, and losing or misplacing the voucher. Lack of money for the top-up amount was not a barrier [40].

### Management and risk mitigation

Fraud risk mitigation measures were put in place since the start of the TNVS. Measures for the paper voucher included specialized printing with security features, spot checks at health facilities and retail outlets, and oversight of the voucher supply chain by Local Government Authorities. Fraud was estimated to be around 10% between 2005 and 2010, dropping to 5% in 2011 [40]. Fraud included theft of voucher booklets and forgery of names on vouchers. No counterfeit paper vouchers were found. Local Government Authorities played a key role in working with the Logistics Contractor to monitoring the paper voucher system and took when necessary punitive action against health facility staff and retailers involved in fraud.

For the electronic voucher a web-based tracking tool was put in place which provided real-time data on e-voucher redemption per clinic and retailer. Additional e-voucher controls were put in place early 2014 after suspicion of fraud. Risk mitigation measures included: tracking of e-vouchers issued beyond normal clinic hours; limiting redemption of e-voucher to the region where they were issued; and capping the number of vouchers that a facility could issue based on estimated RCH attendance [41]. However, these measures were introduced at a very late stage when fraud had already escalated.

Furthermore, contrary to the paper voucher supply chain, Local Government Authorities had significantly less control over the electronic voucher cycle since e-vouchers were generated directly by the facilities. Local Government Authorities were informed by the Logistics Contractor about cases of fraud after the fact so that they could take necessary disciplinary action against involved staff. There was however insufficient ownership by the LGA of the e-voucher system to play an active role in monitoring and prevention of fraud.

### Global and donor objectives

Reliance on external funding made the TNVS vulnerable in several ways. The influence of global and donor objectives, changing from stimulating a sustainable commercial market in ITNs to achieving and maintaining universal coverage, fundamentally changed the design of the programme. Although coverage of vulnerable populations remained the key objective of the TNVS, the secondary objective to stimulate a viable commercial net market did not get the attention it deserved in later years.

Funding availability and target setting by successive donors affected the number of vouchers in circulation. A decrease in the number of vouchers issued had a direct impact on the number of nets bought with a voucher, and subsequently the supply of LLINs by retailers. The electronic voucher had the potential to increase access to a voucher, but due to a design flaw and sub-optimal management a fatal fraud developed. Not including the final two quarters, the number of vouchers issued at RCH clinics was substantially lower than the projected eligible population, indicating that the TNVS mostly did not reach its full capacity. This was partly because understanding of decay of nets in field conditions and the number of nets needed to maintain coverage [42] is recently new and was not sufficiently taken into account in the target setting of the TNVS.

The ultimate decision by DFID to discontinue funding caused the end of a national programme which had been operating well for over a decade. Although the audit report was never disclosed to the Ministry of Health, the heated discussion around the fraud findings brought the voucher scheme into disrepute. This is a shame considering all the successes the programme had achieved. Consequently, the Government of Tanzania was not able to secure new funding for the TNVS. Mid 2016, the Government of Tanzania started a new programme of free distribution of LLINs through RCH facilities with funding from the GFATM and PMI. The public sector will now be responsible for all aspects of the LLIN supply chain management including physical distribution and storage of nets, in addition to registration of beneficiaries.

### Replication in other settings

A key strength of the voucher scheme was that the private sector managed the net supply chain through a wide retail network, thereby alleviating the burden on the public sector to order, transport, stock and distribute bulky nets. Furthermore, the TNVS was instrumental in creating and maintaining continuous access to nets through shops, in both rural and urban areas. This was crucial to keep up LLIN coverage of vulnerable populations in the intermittent period between mass campaigns. The high cumulative redemption rates throughout the lifespan of the programme show that there was a strong demand for subsidized nets even during campaigns. Recent data from the 2015–16 DHS survey in Tanzania indicate that sales of untreated nets in Tanzania are appearing to increase, with the 27.9% of all nets in households sourced from the commercial sector, ranging from 13.9% in rural areas to 54.4% in urban areas [43]. Thus, it does appear that during the period of the TNVS, a ‘net culture’ was established in Tanzania and households are seeking nets from shops when they perceive a need for an additional net.

To date the TNVS is the only national scale voucher scheme that was successfully implemented. A sub-national level programme was operated in Ghana [44], but it never reached national level implementation and was not sustained. Several small schemes operated in other countries, but none came close to national-scale implementation Given the advantages of a voucher scheme, it is surprising that no other country ever took up this form of keep-up distribution of LLINs. By contrast, many countries directly distribute free LLINs through antenatal clinics [1].

### Impact on malaria incidence and mortality rates

Malaria control in general has had a large health impact in Tanzania, particularly after the upscaling of the use of ITNs, including through the TNVS. In 2000, the number of malaria cases in Tanzania was estimated to be 16 million. By 2015, this number dropped to 7.7 million [45]. Tanzania has seen a 55% in decline in all-cause mortality in children under the age of five between 2000 and 2015 [30, 31, 43, 46]. It has been estimated that at least 15% of this decline can be directly attributed to malaria control interventions, not including the impact of reduced malaria burden on ‘indirect’ malaria mortality (deaths in which malaria was a contributing cause) [47, 48].

## Conclusions

The Voucher Scheme was a fundamental component of NATNETS, and played a pivotal role in providing pregnant women and infants with continuous access to LLINs. The programme reached the majority of beneficiaries with vouchers and provided 1.2 million to 1.8 million highly subsidized LLINs per year. Approximately 30% of all (long lasting) insecticide treated nets distributed with public funding in the period 2004–2014 were supplied through the TNVS. It was a unique, innovative and globally influential programme that stimulated strategic thinking about effectively and equitably distributing ITNs, and contributed directly to the evolution of global LLIN policy.

The design of the programme was continually adjusted based on new evidence, programmatic experience, and changing policy targets. As the first of its kind on this scale, the TNVS paved the way and did not have the benefit of learning from other LLIN distribution programmes. Much of the current knowledge on the lifespan of nets and *catch up* and *keep up* strategies was not yet known when the programme was designed. The TNVS substantially increased ownership and use of LLINs amongst vulnerable populations in the entire country, but was never designed or intended to achieve universal coverage. This could only be achieved through the mass distribution of free nets, with the TNVS functioning as the main keep-up mechanism.

The TNVS was a pioneer in harnessing the strength of a PPP to ensure continuous net distribution on a national scale. The TNVS maintained over many years a nationwide retail network of over 7000 retailers which formed the downstream end of the LLIN supply chain. However, the premise of achieving ITN upscaling through a strong commercial supply chain for nets supplemented by subsidized sales to target populations was never achieved. Nevertheless, the private sector was instrumental in maintaining a continuous flow of LLINs throughout the country on behalf of the public sector. Finally, the TNVS represents an outstanding example of a successful public private partnership for a major health intervention.

## Abbreviations

DFID: UK Department for International Development
DMO: District Medical Officers
GFATM: Global Fund to Fight AIDS, TB and Malaria
ITN: insecticide-treated nets
IV: infant voucher
LLINs: long-lasting insecticidal nets
LGA: Local Government Authorities
NATNETS: National Insecticide Treated Nets Strategy
PMI: United States Agency for Aid and Development, President’s Malaria Initiative
PPP: public private partnership
PWV: pregnant woman voucher
TNVS: Tanzania National Voucher Scheme
RCH: reproductive and child health
